# Walking exercise alters pedunculopontine nucleus connectivity in Parkinson’s disease in a dose-dependent manner

**DOI:** 10.3389/fnins.2022.930810

**Published:** 2022-08-09

**Authors:** Jiayue Cai, Aiping Liu, Yuheng Wang, Sun Nee Tan, Taylor Chomiak, Jacqueline Burt, Richard Camicioli, Bin Hu, Martin J. McKeown, Fang Ba

**Affiliations:** ^1^School of Biomedical Engineering, Health Science Center, Shenzhen University, Shenzhen, China; ^2^Division of Neurology, Department of Medicine, The University of British Columbia, Vancouver, BC, Canada; ^3^Department of Electronic Science and Technology, University of Science and Technology of China, Hefei, China; ^4^School of Biomedical Engineering, The University of British Columbia, Vancouver, BC, Canada; ^5^Graduate Program in Neuroscience, The University of British Columbia, Vancouver, BC, Canada; ^6^Department of Clinical Neurosciences, University of Calgary, Calgary, AB, Canada; ^7^Division of Neurology, Department of Medicine, University of Alberta, Edmonton, AB, Canada

**Keywords:** fMRI, functional connectivity, pedunculopontine nucleus, Parkinson’s disease, walking exercise

## Abstract

**Background:**

Gait disturbances are critical motor symptoms in Parkinson’s disease (PD). The mechanisms of gait impairment in PD are not entirely understood but likely involve changes in the Pedunculopontine Nucleus (PPN), a critical locomotion center, and its associated connections. Exercise is universally accepted as helpful in PD, but the extent and intensity of exercise required for plastic changes are unclear.

**Methods:**

Twenty-seven PD subjects participated in a 3-month gait training intervention. Clinical assessments and resting-state functional magnetic resonance imaging were performed at baseline and 3 months after exercise. Functional connectivity of PPN was assessed by combining the methods of partial least squares, conditional dependence and partial correlation. In addition, paired t-tests were used to examine the effect of exercise on PPN functional connectivity and clinical measures, and Pearson’s correlation was used to assess the association between altered PPN functional connectivity and clinical measures.

**Results:**

Exercise significantly improved Unified Parkinson’s Disease Rating Scale-III (UPDRS-III). A significant increase in right PPN functional connectivity was observed after exercise, which did not correlate with motor improvement. However, the decrease in left PPN functional connectivity significantly correlated with the improvement in UPDRS-III and was linearly related to both number of walks and the duration of walks. In addition, exercise induced a significant increase in the laterality of PPN connectivity strength, which correlated with motor improvement.

**Conclusion:**

PPN functional connectivity is modifiable by walking exercise in both a dose-independent (right PPN and laterality of PPN connectivity strength) and dose-dependent (left PPN) manner. The PPN may contribute to pathological and compensatory processes in PD gait control. The observed gait improvement by walking exercise is most likely due to the reversal of the maladaptive compensatory mechanism. Altered PPN functional connectivity can be a marker for exercise-induced motor improvement in PD.

## Introduction

Gait disturbances in Parkinson’s disease (PD), such as decreased stride length, gait variability and especially freezing of gait (FOG), are associated with increased fall risk and hence a significant source of disability (Schaafsma et al., 2003). Falls have devastating impacts on the quality of life of individuals with PD, often trigger a downward spiral of frailty and can lead to depression, social isolation, activity avoidance, and fear of falling (Bloem et al., 1996). Some therapeutic attempts have been proposed to address gait impairment, including different training approaches (Nonnekes et al., 2019; Canning et al., 2020; Cosentino et al., 2020). Nevertheless, gait impairment, including FOG, falls, and postural instability remains largely untreatable in PD (Perez-Lloret et al., 2014). New therapeutic approaches and finding potentially new targets for intervention are desperately needed.

The neuropathological substrates underlying postural and gait impairment in PD are poorly understood. Pre-motor, primary, and supplementary motor cortical areas and the cerebellum and basal ganglia modulate brainstem structures. The brainstem acts as a central communication hub in locomotion control. In PD, the role of the brainstem may include both compensatory and pathological mechanisms (Hacker et al., 2012). One important brainstem structure in gait control is the mesencephalic locomotor center (MLC), which generates postural responses and influences equilibrium, balance and gait (Tattersall et al., 2014). A vital component of the MLC is the pedunculopontine nucleus (PPN), a predominant cholinergic structure in the brainstem with widespread connectivity to other brain regions. The PPN receives direct glutamatergic inputs from the motor cortex and GABAergic from substantial nigra, globus pallidus internus (GPi), subthalamic nucleus (STN), and deep nuclei of the cerebellum. Ascending efferent projections from the PPN target GPi, substantia nigra pars compacta, and thalamus; descending efferent projections target pontine and medullary reticular formation and spinal cord vital for control of muscle tone and locomotion. Alterations in PPN microstructural integrity are associated with gait impairment and postural instability and may predict the development of these problems in PD (Craig et al., 2020). In addition, alternations in PPN structural connectivity are associated with FOG and attentional control during dual-task gait (Fling et al., 2013, 2014; Mancini et al., 2015). Functional connectivity studies suggest that FOG patients may have significantly more robust connectivity between the PPN-supplemental motor area (SMA), reflecting possibly maladaptive compensatory mechanisms and a reorganization of functional communication within the locomotor network (Fling et al., 2014). Thus, one may speculate that the role of the PPN may contribute to both pathological and compensatory processes in PD gait control (Holiga et al., 2015; Craig et al., 2020).

As the PPN is affected in PD, there has been intense interest in modulating PPN activity, but results have been inconsistent (Wang et al., 2016). Acetylcholinesterase inhibitors affect the PPN, but the effects are modest (Henderson et al., 2016). Multiple PPN-deep brain stimulation (DBS) studies have suggested possible clinical improvement in patients with PD. However, results have varied (Stefani et al., 2007; Khan et al., 2011). Reasons for this response variability may be difficulties determining precise DBS electrode placement and variability of structural and functional connectivities to and from the PPN. A recent study has demonstrated that Galvanic Vestibular Stimulation (GVS) in PD subjects can modify PPN connectivity, but not in controls, with changes in functional connectivity correlating with Unified Parkinson’s Disease Rating Scale motor score (UPDRS-III) (Holiga et al., 2015; Cai et al., 2018), which is a standardized scale to assess PD patients’ motor performance and a widely applied index of disease severity, with higher scores indicating more severe motor symptoms.

Behaviorally, exercise, physiotherapy and other measures targeting stepping-in-place are effective interventions for PD gait impairment and preventing falls (Chomiak et al., 2017; Xian et al., 2018). A recent study has demonstrated that aerobic exercise increased functional connectivity of the sensorimotor circuit and functional connectivity in the right frontoparietal network and improved cognitive control (Johansson et al., 2022). However, the effects of these interventions on PPN connectivity are unknown. In the current study, our goal is not to demonstrate the efficacy of walking in improving PD, as we believe that the beneficial effects of exercise are well established (da Silva et al., 2016; Burt et al., 2019), and this would require a control condition. Instead, our goal is to determine if the *amount of exercise affects the extent of functional connectivity changes in PD*. Since there is increasing recognition that several cortical and subcortical regions may be necessary for gait difficulties in PD (Fling et al., 2014), we specifically employed a hypothesis-driven approach. We examined PPN connectivities because it is a potential target in DBS. Such a hypothesis-driven approach prevents the need to correct massive multiple comparisons required for a fully exploratory approach. Careful care was taken to ensure robust activation from PPN structures by analyzing the data in native space (without registration to a template) and utilizing subject-specific weightings of voxels within the PPN region.

## Materials and methods

### Subjects

**FIGURE 1:**
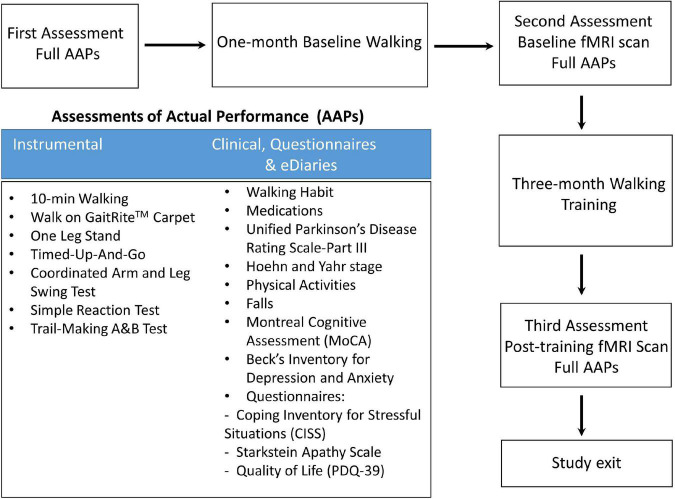
Flowchart for the study protocol.

Twenty-seven gait impaired PD subjects (Table 1) with mild to moderate disease (Hoehn and Yahr stage I–III) and optimally treated with PD medications were recruited from the Pacific Parkinson’s Research Centre at the University of British Columbia (UBC). All patients provided written informed consent before participation. The Institutional Review Board of the UBC approved the study protocol. The study design and protocol are shown in Figure 1.

**TABLE 1:** Demographic and clinical information on PD patients.

Clinical characteristics	Statistics (Mean ± standard deviation)
Gender	17 Males, 10 Females
Age	64.5 ± 8.1
Disease duration (years)	4.9 ± 4.0
UPDRS III	23.9 ± 11.3
H&Y	1.4 ± 0.5
FES-I	20.1 ± 5.7
FOG-Q	2.8 ± 4.0
MoCA	27.0 ± 3.1
Total number of walks	29.8 ± 17.2
Total length of training (min)	1471.7 ± 849.4
Average walking (m/min)	73.1 ± 18.5
Total walking distance (km)	112.4 ± 71.7

Values are given as mean ± standard deviation. UPDRS III, Unified Parkinson’s Disease Rating Scale-Part III; H&Y, Hoehn and Yahr stage; FES-I, Falls Efficacy Scale International; FOG-Q, Freezing of Gait Questionnaire; MoCA, Montreal Cognitive Assessment.

### Intervention

The walking exercise intervention started with 1 month of walking conducted at home to determine each subject’s baseline overall pattern of home exercise and gait features while allowing participants to familiarize themselves with the intervention. After that, participants received a 3-month home-based walking training. They conducted regular walking exercise without listening to music, walking exercise with music all the time, or walking exercise with music contingent upon the amplitude of walking steps. An iPod touch™ (4th generation) sensor system device was used for music via Bluetooth headphones and for assessing stride length. During the training, participants were instructed to walk at a minimum of 20 min/walk, three times/week or 60 min/week, at their preferred pace. In addition, they were required to upload their walking files weekly via a wireless internet connection to an online database. The walking exercise intervention has been shown feasible in PD patients (Burt et al., 2019), improving PD gait disorders (Xian et al., 2018) that included step automaticity while dual-tasking (Chomiak et al., 2017). For this study, we pooled all 27 PD participants to increase the statistical power and assessed the effect of “general” walking exercise on functional connectivity changes.

All 27 PD patients completed Assessments of Actual Performance (AAPs, Figure 1) from the first visit to the last for pre- and post- intervention comparison. fMRI brain scans were performed at baseline and 3 months post-training. Patients were in medication ON state during the clinical assessment and MRI scan. Five patients did not have a repeat UPDRS-III at follow-up and were subsequently excluded from the UPDRS-related analysis.

### fMRI data acquisition and preprocessing

**FIGURE 2:**
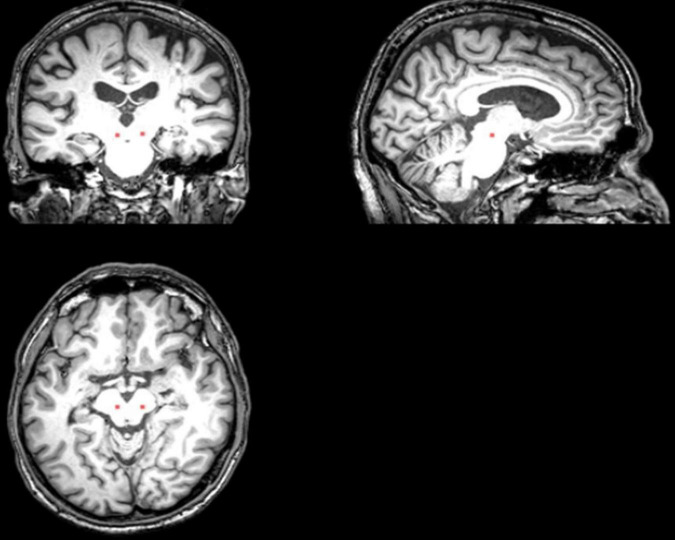
The placement of the PPN ROI on the T1 sequence (Cai et al., 2018).

Resting-state data were collected using a 3T scanner (Philips Achieva 3.0T R3.2; Philips Medical Systems, Netherlands) equipped with an 8-channel head-coil. High-resolution T1 weighted anatomical images were acquired using the following parameters: a repetition time of 1,970 ms, echo time of 3.9 ms, inversion time of 1,100 ms and flip angle of 15°. The bilateral PPN regions of interest (ROIs) were manually drawn on the T1 image at the level of the superior cerebellar decussation between the medial lemniscus and superior cerebellar peduncle (Aravamuthan et al., 2007), presumably including the PPN and cuneiform nucleus (Figure 2). BOLD contrast T2*-weighted images were obtained using a gradient-echo EPI sequence with the repetition time of 1,985 ms, echo time of 37 ms, flip angle of 90°, a field of view of 240 mm, matrix size of 128 × 128, and pixel size of 1.9 × 1.9 mm. The scan duration of each functional run was 8 min at the resting state.

Analysis of Functional NeuroImages (AFNI)^1^ and Statistical Parametric Mapping (SPM8)^2^ software packages were used for fMRI data preprocessing, including despiking, slice timing correction, 3D isotropic correction, and motion correction using rigid body alignment to correct any significant head movements during the scan. All preprocessing steps were performed in the individual subject’s native space to avoid possible registration errors induced by warping the data to a common template. First, FreeSurfer was utilized for standard brain parcellation on T1-weighted images. Co-registration between parcellated structural images and functional data was done in FMRIB Software Library (FSL)^3^. Next, nuisance signals, such as head movement parameters, their temporal derivatives and their squares, white-matter signal and cerebrospinal fluid signal, were regressed from the preprocessed data. Finally, fMRI signals were temporally detrended, followed by spatial smoothing with a 6 × 6 × 6 FWHM Gaussian kernel and a bandpass filtering at 0.01–0.08 Hz.

The inverse transformation of registering functional data to structural images was utilized to register PPN voxels drawn on the T1-weighted image to the functional space. To account for possible partial volume effects, both PPN voxels and their neighboring voxels were included in the analysis. In addition, a separate motion correction was performed within the brainstem, accounting for possible brainstem motions independent of the rest of the brain.

### Pedunculopontine nucleus functional connectivity analyses

Functional connectivity analysis was performed between two PPN ROIs (i.e., left and right PPN) and 80 cortical/subcortical ROIs (Table 2). The analyses were conducted in three steps: the first step is to select a candidate set of cortical/subcortical ROIs that significantly covaried with PPN voxels by using partial least squares (PLS) (Cai et al., 2018), followed by a functional connectivity network structure learning step by applying the PC_*fdr*_ algorithm (Li and Wang, 2009) to PPN ROIs and PLS-selected cortical/subcortical ROIs, and finally estimate functional connectivity coefficients by computing partial correlations.

**TABLE 2:** The PLS-selected ROIs (bold, italic) from the 80 ROIs used in this study.

Index	Name	Index	Name
1	***Left-Cerebellum-Cortex***	39	***Right-Cerebellum-Cortex***
2	***Left-Thalamus-Proper***	40	***Right-Thalamus-Proper***
3	***Left-Caudate***	41	***Right-Caudate***
4	***Left-Putamen***	42	***Right-Putamen***
5	***Left-Pallidum***	43	***Right-Pallidum***
6	Left-Hippocampus	44	***Right-Hippocampus***
7	Left-Amygdala	45	***Right-Amygdala***
8	***Left-Accumbens-area***	46	***Right-Accumbens-area***
9	ctx-lh-parahippocampal	47	ctx-rh-parahippocampal
10	***ctx-lh-insula***	48	***ctx-rh-insula***
11	***ctx-lh-superiorfrontal***	49	***ctx-rh-superiorfrontal***
12	***ctx-lh-rostralmiddlefrontal***	50	***ctx-rh-rostralmiddlefrontal***
13	***ctx-lh-caudalmiddlefrontal***	51	***ctx-rh-caudalmiddlefrontal***
14	***ctx_lh_G_front_inf-Opercular***	52	***ctx-rh-G_front_inf-Opercular***
15	***ctx_lh_G_front_inf-Orbital***	53	***ctx-rh-G_front_inf-Orbital***
16	***ctx_lh_G_front_inf-Triangul***	54	***ctx-rh-G_front_inf-Triangul***
17	***ctx-lh-lateralorbitofrontal***	55	***ctx-rh-lateralorbitofrontal***
18	***ctx-lh-medialorbitofrontal***	56	***ctx-rh-medialorbitofrontal***
19	***ctx-lh-caudalanteriorcingulate***	57	***ctx-rh-caudalanteriorcingulate***
20	***ctx-lh-rostralanteriorcingulate***	58	***ctx-rh-rostralanteriorcingulate***
21	ctx-lh-entorhinal	59	ctx-rh-entorhinal
22	***ctx-lh-inferiortemporal***	60	***ctx-rh-inferiortemporal***
23	***ctx-lh-middletemporal***	61	***ctx-rh-middletemporal***
24	***ctx-lh-superiortemporal***	62	***ctx-rh-superiortemporal***
25	***ctx_lh_G_occipital_sup***	63	***ctx-rh-G_occipital_sup***
26	***ctx_lh_G_oc-temp_lat-fusifor***	64	***ctx-rh-G_oc-temp_lat-fusifor***
27	***ctx_lh_G_oc-temp_med-Lingual***	65	***ctx-rh-G_oc-temp_med-Lingual***
28	***ctx-lh-inferiorparietal***	66	***ctx-rh-inferiorparietal***
29	***ctx-lh-postcentral***	67	***ctx-rh-postcentral***
30	***ctx-lh-posteriorcingulate***	68	***ctx-rh-posteriorcingulate***
31	***ctx-lh-precuneus***	69	***ctx-rh-precuneus***
32	***ctx-lh-cuneus***	70	***ctx-rh-cuneus***
33	***ctx-lh-superiorparietal***	71	***ctx-rh-superiorparietal***
34	***ctx_lh_G_pariet_inf-Angular***	72	***ctx-rh-G_pariet_inf-Angular***
35	***ctx_lh_G_pariet_inf-Supramar***	73	***ctx-rh-G_pariet_inf-Supramar***
36	***L_M1***	74	***R_M1***
37	***L_SMA_proper***	75	***R_SMA_proper***
38	***L_Pre_SMA***	76	***R_Pre_SMA***
39	***L_PMd***	78	***R_PMd***
40	***L_PMv***	80	***R_PMv***

First, PLS (Cai et al., 2018) was performed between the PPN voxel-based dataset and cortical/subcortical-based ROI dataset. PLS is a well-known statistical method for analyzing the covariance relationship between two data sets. It decomposes each data set into a new set of latent variables (also called components) and maximizes the covariance between the two data sets in terms of latent variables.

By applying PLS, the two data sets correspond to the 80-ROI dataset and the PPN voxel dataset. The general PLS model is:


(1) $$\text{X}=\text{U}\,W^{T}+\text{A}$$

(2) $$\text{Y}=\text{V}\,Z^{T}+\text{B}$$

where *X* and *Y* are matrices of the ROI dataset and PPN voxel dataset, with the dimensions of *n* × *p* and *n* × *q* respectively, where *n* is the number of time points, *p* (= 80) is the number of ROIs (subject-independent), and *q* is the number of PPN voxels (subject-dependent); *U* and *V* are *n* × *c* matrices of latent variables (also called components), with *c* corresponding to the number of components; *W* and *Z* are loading matrices with the dimensions of *p* × *c* and *q* × *c*, respectively, *A* and *B* are error terms.

The loadings of the dominant component of the ROI dataset (i.e., the first column of *W*) were interrogated to determine if they were significantly different from zero across subjects. In this way, the ROIs of which the time courses significantly covaried with the time courses of PPN voxels were selected as the candidate set of brain regions. In addition, the dominant component of the PPN voxel dataset (i.e., the first column of *V*) was utilized to represent the PPN signal in the subsequent functional connectivity analysis.

Second, functional connectivity network structure was assessed between the PPN and PLS-selected ROIs using the PC_*fdr*_ algorithm (Li and Wang, 2009). PC_*fdr*_ algorithm is a Bayesian network learning approach that enables the control of the false discovery rate (FDR). It is suitably modified from the PC algorithm, which estimates the existence of edges in a graph by determining the conditional dependence relationships between nodes. While the original PC algorithm controls the type I error rate, the PC_*fdr*_ algorithm embedded the FDR control procedure into the PC algorithm to curb the FDR of the learned network structure, which was set to be 0.05. In this step, the functional connectivity of the PPN is estimated.

Finally, the connection coefficients of PPN functional connectivity were evaluated by computing partial correlation coefficients on a subject-by-subject basis. We utilized a “common structure” approach (Chen et al., 2017), which imposes the same connectivity network structure on each subject while allowing connectivity coefficients to be different across subjects.

### Statistical analyses

To investigate the effect of walking exercise on PPN functional connectivity, we quantified three metrics: (1) individual PPN functional connectivity, which is defined as the absolute value of the calculated individual connectivity coefficient; (2) overall PPN functional connectivity, which is defined as the sum of individual PPN functional connectivity; (3) laterality of PPN connectivity strength, which is defined as the magnitude difference between averaged right and left PPN functional connectivity. A paired *t*-test was utilized to compare the overall left/right PPN functional connectivity, laterality of PPN connectivity strength and UPDRS-III score between pre-exercise and post-exercise. Associations between PPN functional connectivity (including overall left/right PPN functional connectivity and laterality of PPN connectivity strength), UPDRS-III score and walking metrics on all participants were investigated using Pearson’s correlation. FDR correction was applied for multiple comparisons. Statistical significance was set at *p* < 0.05.

## Results

**FIGURE 3:**
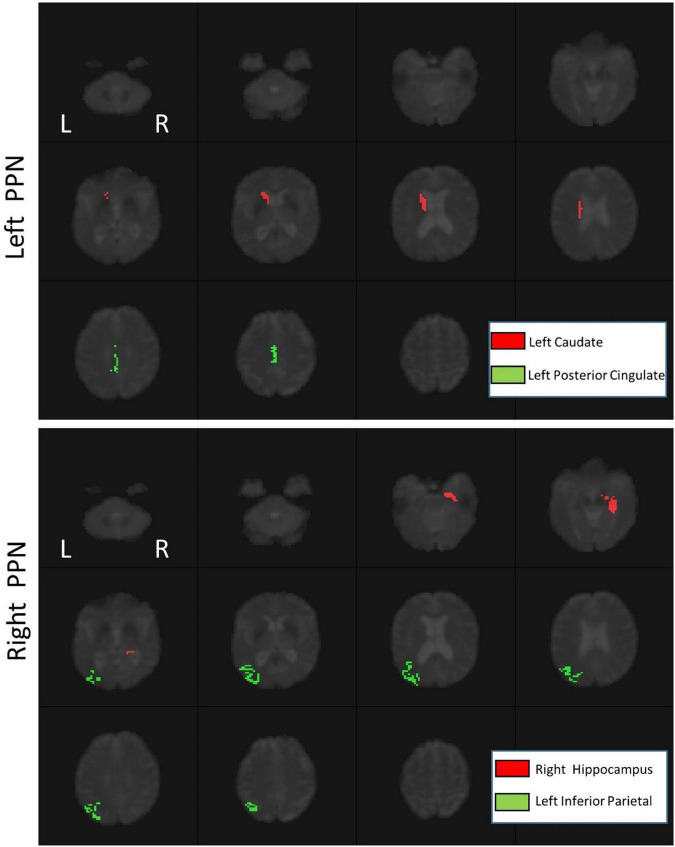
The functional connectivity of the left PPN (top) and right PPN (bottom).

Using PLS, the signals of 74 ROIs significantly covaried with the signals of PPN voxels (Table 2, *p* < 0.01). In addition, there was significant functional connectivity between the left caudate and left posterior cingulate and the left PPN (*p* < 0.05). For the right PPN, significant functional connectivities with the right hippocampus and left inferior parietal were found (*p* < 0.05), as shown in Figure 3.

### Exercise improves motor performance

**FIGURE 4:**
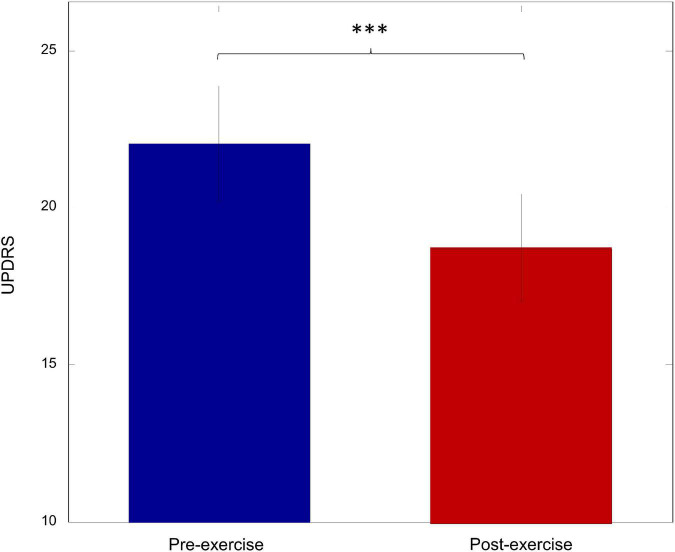
The UPDRS score for pre-exercise and post-exercise (***p < 0.005).

To investigate the effect of walking exercise on the motor performance of PD patients, the UPDRS-III score was compared between pre-exercise and post-exercise. As shown in Figure 4, there was a significant decrease in the UPDRS-III score after exercise (*p* = 0.003). This suggests that the motor performance was significantly improved by walking exercise.

### Pedunculopontine nucleus functional connectivity changes after exercise

**FIGURE 5:**
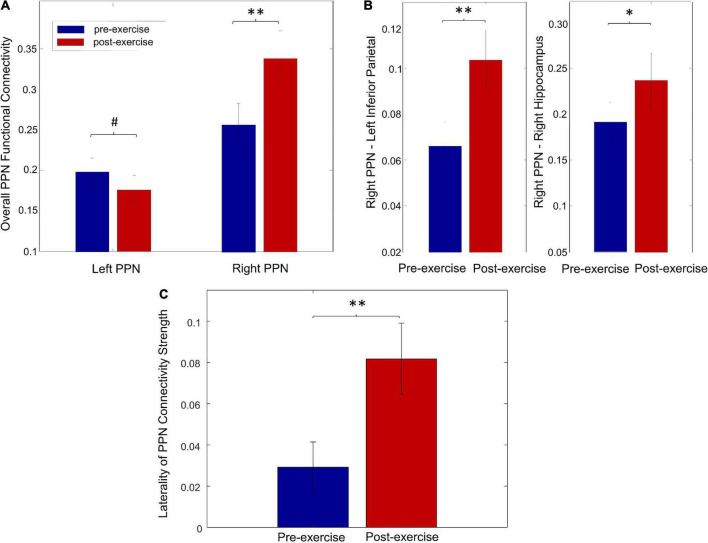
Exercise-induced changes on PPN functional connectivity. **(A)** The overall functional connectivity of the left and right PPN for pre-exercise and post-exercise. **(B)** The individual functional connectivity of the right PPN for pre-exercise and post-exercise. **(C)** The laterality of PPN connectivity strength for pre-exercise and post-exercise (^#^*p* > 0.1, *p < 0.1, **p < 0.05).

Exercise significantly increased the overall right PPN connectivity (Figure 5A, *p* = 0.02) but showed a trend of decreasing the overall PPN connectivity on the left side (Figure 5A, *p* = 0.83). Specifically, the connectivity between the right PPN and left inferior parietal was significantly increased (Figure 5B, *p* = 0.03), and a trend of increase in connectivity was found between the right PPN and right hippocampus (Figure 5B, *p* = 0.07). No significant changes were found in the individual left PPN functional connectivity. In addition, a significant increase in the laterality of PPN connectivity strength was observed (Figure 5C, *p* = 0.02).

### Pedunculopontine nucleus functional connectivity correlates with disease severity

**FIGURE 6:**
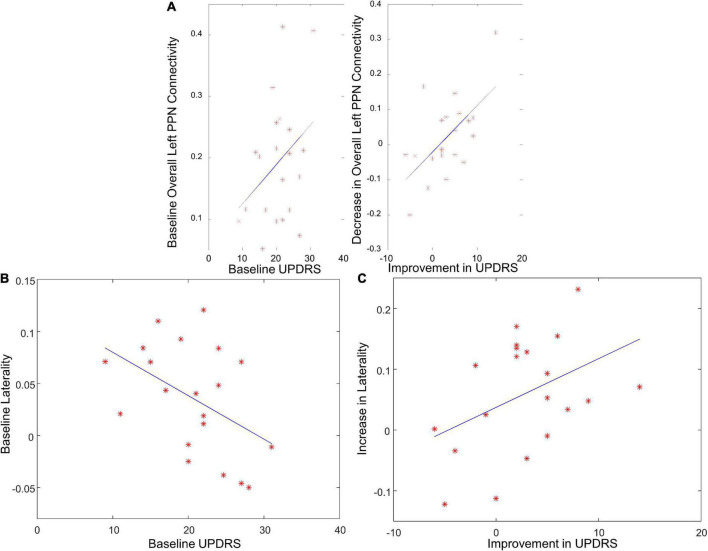
The correlation between PPN functional connectivity and UPDRS score. **(A)** The correlation between overall left PPN functional connectivity and UPDRS score at baseline, and between the decrease in overall left PPN functional connectivity and UPDRS improvement. **(B)** The correlation between the laterality of PPN connectivity strength and UPDRS score at baseline. **(C)** The correlation between the increase in laterality of PPN connectivity strength and UPDRS improvement.

The overall functional connectivity of left PPN positively correlated with the baseline UPDRS-III score (Figure 6A, *r* = 0.36, *p* = 0.05), suggesting that PD patients with less disease severity could have lower overall left PPN functional connectivity. The laterality of PPN connectivity strength negatively correlated with the UPDRS-III score at baseline (Figure 6B, *r* = –0.45, *p* = 0.04), suggesting that PD patients with less disease severity could exhibit larger laterality of PPN connectivity strength. There was a trend of a decrease in overall left PPN functional connectivity after exercise (Figure 5A). Such a decrease associated with exercise was significantly correlated with the improvement in the UPDRS-III score (Figure 6A, *r* = 0.63, *p* = 0.001). In contrast, the overall right PPN functional connectivity was significantly increased after exercise (Figure 5A). There was no clear correlation between the increase in overall right PPN functional connectivity and the improvement in UPDRS-III score; however, individuals with lower baseline UPDRS-III scores showed a higher magnitude of increase in overall right PPN functional connectivity (not significant). The laterality of PPN connectivity strength increased significantly after exercise (Figure 5C, *p* = 0.02). Such increase correlated with the improvement in UPDRS-III (Figure 6C, *r* = 0.42, *p* = 0.04).

### Pedunculopontine nucleus functional connectivity correlates with walking metrics

**FIGURE 7:**
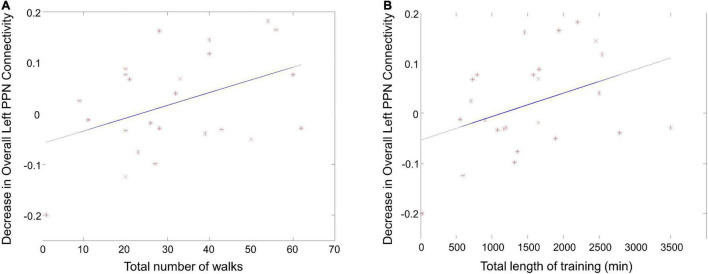
The correlation between PPN functional connectivity and walking metrics. **(A)** The correlation between the decrease in overall left PPN functional connectivity and the total number of walks. **(B)** The correlation between the decrease in overall left PPN functional connectivity and the total length of training.

To examine the dose-response relationship of the exercise, the correlation between overall left/right PPN functional connectivity and walking metrics were analyzed. The decrease in the overall left PPN functional connectivity correlated with the total number of walks (Figure 7A, *r* = 0.43, *p* = 0.02) and the total length of training (Figure 7B, *r* = 0.39, *p* = 0.03), suggesting that PPN functional connectivity was affected by exercise in a dose-dependent manner.

## Discussion

We have shown that walking exercise affects PPN functional connectivity in PD in both a dose-dependent and dose-independent manner. Left and right PPN functional connectivities were different at baseline. We found that the left caudate and left posterior cingulate significantly covaried with the left PPN, and the right hippocampus and left inferior parietal lobule covaried with the right PPN. The left and right PPN connectivities were differently affected by exercise. Although not the primary goal of this study, we demonstrated that walking exercise improved overall UPDRS-III PD motor scores. We observed that the left PPN functional connectivity changes and the change in the laterality of PPN connectivity strength after exercise correlated with improvement in UPDRS-III.

Previously, altered PPN connectivity has been demonstrated chiefly with neuromodulation strategies. Unilateral PPN-DBS during self-paced lower limb movements results in increased regional cerebral blood flow in interconnected structures of the cerebello-thalamo-cortical circuit, including the PPN region (Ballanger et al., 2009). Chronic low-frequency PPN-DBS can modify brain connectivity in FOG, reducing corticopontine “overactivity” seen in the pre-stimulation period (Schweder et al., 2010). We have previously shown that GVS modulates PPN functional connectivity in PD patients (Cai et al., 2018).

Our prior GVS study, which used a similar approach for inferring PPN functional connectivity, demonstrated significantly reduced connectivity of the left PPN in PD subjects with mild to moderate disease (Cai et al., 2018). After exercise, we found significant increases in overall right PPN functional connectivity, more or less independent of disease severity. In contrast, before the intervention, the functional connectivity of the left PPN *positively* correlated with the baseline UPDRS-III score, so even though overall left PPN connectivity was reduced in the PD population, greater connectivity was seen with greater disease severity. There are two potential explanations for this apparent paradox. One is that the left PPN connectivity represents a compensatory response that increases with disease severity. Note that with a compensatory mechanism, reduction of disease effects (as evidenced by the improvement in behavioral measures) will also decrease compensatory mechanisms. Another relates to “neural efficiency,” whereby exercise results in less co-activation of ancillary brain regions, presumably due to more efficient recruitment of necessary resources to complete the task of ambulation (Nishiguchi et al., 2015). In contrast, with the right PPN, the functional connectivity was increased after exercise, but this did not correlate with changes in overall motor function. While it is still unclear whether there is a dominant PPN in humans, especially in PD gait-impaired individuals, our result further supports an asymmetry in PPN connectivity strength, as previously suggested (Cai et al., 2018).

Several studies have demonstrated altered connectivity between left and right PPN. For example, in normal control individuals, faster and typical imagery of gait conditions revealed substantial activation of left PPN (Karachi et al., 2010; Baudrexel et al., 2011; Fling et al., 2014). In PD, asymmetry of PPN connectivity had been shown to relate to FOG. For instance, the volume of white matter tracts emanating from the PPN is diminished in the right hemisphere in PD patients with FOG, resulting in a higher right-left PPN white matter asymmetry in FOG (Peterson et al., 2015). Such PPN white matter asymmetry was seen to be related to measures of inhibition (Fling et al., 2013). In PD patients with FOG, reduced white matter connectivity can be seen from the PPN to the cerebellar locomotor regions, thalamus, and multiple regions of the frontal and prefrontal cortex only in the right hemisphere of freezers (Fling et al., 2013). Functional connectivity in postural instability gait difficulty subgroups has increased functional connectivity between the left PPN and the SMA-proper. At the same time, the right PPN is “hyper-connected” to the right premotor cortex and left M1. Tremor dominant subgroups have increased functional connectivity between the left PPN and the left premotor cortex, pre-SMA and SMA (Vervoort et al., 2016). Greater functional connectivity between the SMA and MLC can be positively correlated with freezing severity in FOG (Fling et al., 2014). The pattern of such enhanced connectivity does not appear to play a functional compensatory role but instead may contribute to FOG.

The left hemisphere is usually dominant in humans; left hemisphere-lateralization of the PPN tract volume in FOG patients is positively correlated with poorer performance on cognitive tasks (Stroop task) (Peterson et al., 2015). Human gait is a complex motor task requiring attention to various environmental changes, shifting attention to avoid trips/falls, and timely recovering from inevitable postural perturbations. The PPN is an important cholinergic structure. The connectivity change from the walking exercise can indicate that brain plasticity for gait control is partially through cognitive involvement through the left PPN connections to the left caudate and left cingulate. From an anatomical perspective, the caudate is an essential structure of locomotion. Direct projections between the PPN and caudate nucleus are evident in animal models (Saper and Loewy, 1982; Nakano et al., 1990), and it has been shown that DBS or lesioning PPN can influence striatal dopamine and acetylcholine activity (Jenkins et al., 2002; Forster and Blaha, 2003). We have previously shown that microstructural alterations in the caudate nucleus are correlated with PD gait abnormalities (Surkont et al., 2021). A reduced dopaminergic function in the caudate nucleus has been shown to associate with the development of postural and gait impairment (Kim et al., 2018, 2019; Craig et al., 2020). The cingulate gyrus also plays an essential role in integrating sensorimotor feedback involving the medial motor strip and other brainstem nuclei, particularly in the dorsal pons (Jha et al., 2017).

As with many previous studies focusing on FOG, it is difficult to compare these results directly with ours, as the group of subjects described in this report have relatively mild disease without FOG. However, one should note that even mild PD is associated with an altered gait, and the asymmetry of PPN connectivity is already evident (Cai et al., 2018), as shown in the current study. In addition, the walking exercise modified the PPN connectivity in a dose-dependent manner. This finding suggests that exercise dose may be an essential parameter for facilitating activity-dependent functional connectivity changes associated with improved motor performance. There is minimal data on the effects of different amounts of exercise on PD patients. Fisher et al. reported dose-dependent benefits of exercise and that high-intensity exercise could normalize corticomotor excitability in early PD. The improved performance was also accompanied by alterations in corticomotor excitability as measured through transcranial magnetic stimulation (Fisher et al., 2008). In addition, van der Kolk et al. have observed a correlation between the increased dose in physical fitness (using VO_2_ max as a surrogate marker) and the decrease in MDS-UPDRS motor scores tested during medication off state (van der Kolk et al., 2019). We demonstrated that PPN functional connectivity change is modified by walking exercise in a dose-dependent manner. The associated improvement in motor function provides additional evidence for how exercise can positively impact PD.

One of the advantages of this study is that we performed analyses keeping each subject’s data in their original space without warping the data to a common template. We are skeptical of fMRI studies suggesting robust activation from brainstem structures (e.g., PPN) when data are spatially transformed to a template, given the significant registration errors that can occur to small brainstem nuclei during whole-brain registration (Ng et al., 2009). In addition, we utilized PLS to find the combination of PPN voxels on a subject-by-subject basis that maximally corresponded with other ROIs. In effect, the dominant component of PLS represents a subject-specific spatial filter to “focus” the activity that is maximally covaried with other ROIs.

There are a few limitations to our study. First, we studied a relatively small number of PD patients. However, by carefully selecting the PPN voxels via PLS on a subject-by-subject basis, we expect to significantly enhance our effect size, thus increasing our statistical power. Our study only examined a representative group of typical PD patients with mild to moderate disease. Third, we did not include freezers. In severe disease, degeneration in the PPN itself may prevent modulation of its connectivity. In addition, by combining structural connectivity studies, for instance, with tractography, we may be able to establish imaging biomarkers to predict responders from non-responders to treatment interventions. Finally, we did not have a non-disease control group. However, our goal here is not to add to the already ample evidence showing the beneficial effects of exercise, mainly walking. Instead, our goal here is to demonstrate the dose-response of such an intervention.

## Conclusion

The current study observed that PPN functional connectivity is modifiable by walking exercise in both a dose-independent manner (right PPN and laterality of PPN connectivity strength) and a dose-dependent (left PPN) manner. Our results provide additional evidence of altered functional connectivity of the locomotor network in PD patients and further support evidence of lateral PPN connectivity strength early in the disease course. The PPN may contribute to pathological and compensatory processes in PD gait control. The observed gait improvement by walking exercise is most likely due to the reversal of the maladaptive compensatory mechanism. Altered PPN functional connectivity can be a marker for exercise-induced motor improvement in PD.

## Data availability statement

The raw data supporting the conclusions of this article will be made available by the authors upon request, without undue reservation.

## Ethics statement

The studies involving human participants were reviewed and approved by the Institutional Review Board of University of British Columbia. The patients/participants provided their written informed consent to participate in this study.

## Author contributions

JC contributed to the conceptualization, methodology, and software of this project. JC, AL, and YW performed the analysis of data. JC and YW conducted the result validation. ST and TC contributed to the acquisition of data. JB, RC, BH, MM, and FB performed the interpretation of data. JC and FB conducted the writing of the original draft. MM, AL, and YW performed the review and editing of the manuscript. MM and BH contributed to the funding acquisition. All authors contributed to the article and approved the submitted version.

## Footnotes

1
http://afni.nimh.nih.gov/afni/

2
http://www.fil.ion.ucl.ac.uk/spm

3
http://www.fmrib.ox.ac.uk/fsl
